# Cytogenetics of the Porthole Shovelnose Catfish, *Hemisorubim platyrhynchos* (Valenciennes, 1840) (Siluriformes, Pimelodidae), a widespread species in South American rivers

**DOI:** 10.3897/CompCytogen.v7i2.4901

**Published:** 2013-04-25

**Authors:** Ana Cláudia Swarça, Sebastian Sanchez, Ana Lucia Dias, Alberto Sergio Fenocchio

**Affiliations:** 1 Departamento de Histologia, CCB, Universidade Estadual de Londrina, CEP 86051-970, Caixa Postal 6001, Londrina, Paraná, Brazil; 2 Instituto de Ictiología del Nordeste. Facultad de Ciencias Veterinarias. Universidad Nacional del Nordeste. Sargento Cabral 2139. 3400 Corrientes, Argentina; 3 Departamento de Biologia Geral, CCB, Universidade Estadual de Londrina, CEP 86051-970, Caixa Postal 6001, Londrina, Paraná, Brazil; 4 Universidad Nacional de Misiones, Departamento de Genética, Félix de Azara 1552. 3300 Posadas, Misiones, Argentina

**Keywords:** *Hemisorubim platyrhynchos*, Pimelodidae, Cytogenetics

## Abstract

*Hemisorubim platyrhynchos* is a medium- to large-sized pimelodid catfish distributed along several river basins of the Neotropical Region, noteworthy for representing an important fishery source. In this work, *Hemisorubim platyrhynchos* from three isolated populations were cytogenetically analyzed. The karyotype shows a diploid number of 2n=56 chromosomes comprising 22m, 16sm, 10st, 8a (FN=104). NORs detected by AgNO_3_ were located in the terminal regions of the short arm of a st chromosome pair, as confirmed by CMA_3_ and FISH using an 18S rDNA probe. C-banding revealed a small amount of heterochromatin in chromosomes, including the NORs, and one biarmed pair that showed conspicuous positive bands on both arms. This fact was also evidenced when using other banding techniques, such as RE (*Alu*I), and indicates that this pair constitutes a species-specific cytogenetic marker.

## Introduction

*Hemisorubim platyrhynchos* (Valenciennes, 1840), popularly called “jurupoca” or porthole shovelnose catfish, is a pimelodid fish inhabiting the deeper and slow-moving sections of large South American rivers (Burgess 1989, Froese and Pauly 2012). This species is the sixth largest pimelodid of the Pantanal region/Brazil (Penha et al. 2004) and is considered an important species in the fisheries of the Paraná River basin (Agostinho et al. 1995). Its body shape and color pattern are adapted to the muddy bottom where it stays. It feeds on benthic organisms and fish (Froese and Pauly 2012). Breeding and sexing information have not been reported. The extensive exploitation of its populations due to overfishing could affect its genetic variability, which is still poorly known.

The family Pimelodidae represents one of the most specious catfish groups, however relationships among species of this group still remain as unanswered questions; however, it seems self-evident that they share certain characteristics (Nelson 2006, Ferraris 2007). Some authors has been divided this family in “groups” i.e. *Calophysus* Müller and Troschel in Müller 1843, *Pimelodus* LaCépède, 1803 and “Sorubiminae” (de Pinna 1998).

From a cytogenetic point of view some reports show that these groups could also share cytogenetic characteristics, supporting additionally the classification above mentioned (Swarça et al. 2007, Sànchez et al. 2010, Carvalho et al. 2011).

*Hemisorubim platyrhynchos* is a monotypic species that belongs to the family Pimelodidae, however, it is considered one of the “sorubimine catfishes”, an informal group of catfish that comprises other genera such as *Sorubim* Cuvier, 1829, *Pseudoplatystoma* Bleeker, 1862, and *Brachyplatystoma* Bleeker, 1862 (Lundberg and Akama 2005).

Until now only one population of *Hemisorubim platyrhynchos* of the Parana River (Brazil) has been cytogenetically studied and has had its diploid number, AgNORs location and C-banding reported (Martins-Santos et al. 1996).

The objective of the present study was to describe the karyotypic structure of specimens from three populations of *Hemisorubim platyrhynchos* aiming to characterize andcompare the obtained results with the available cytogenetic data on this and other related species.

## Material and methods

Fifteen specimens of *Hemisorubim platyrhynchos* consisting of 8 males (m) and 5 females (f), caught in the Parana River (Corrientes State, Argentina) and 2 specimens of undetermined sex from the Miranda River (Mato Grosso do Sul State, Brazil) were cytogenetically analyzed. The sampling sites in the Paraná River were: Ituzaingó (2 m), Itá Ibaté (2 f - 3 m), Yahapé (1 m), Puerto Abra (1 f), and Corrientes (2 f / 2 m) (Corrientes Province). Mitotic chromosome preparations were obtained according to the technique described by Foresti et al. (1993) for some specimens of Paraná River/Argentina and from blood culture for specimens of the Miranda River/Brazil (Fenocchio and Bertollo 1988). The specimens were deposited in the collection of the Laboratory of the Instituto de Ictiología del Nordeste, Universidad Nacional del Nordeste/Argentina. Nucleolus organizer regions (Ag-NORs) were revealed by the silver-staining method (Howell and Black 1980) and C-banding was performed according to Sumner (1972). Restriction endonucleases were used according to Sánchez et al. (1990) with some modifications, i.e., *Alu*. I was used a concentration of 0.3 U/μL and the chromosome preparations were treated at 37°C for 4h. Chromosome staining with Chromomycin A_3_ (CMA_3_), a C-G specific fluorochrome, was applied according to the description provided by Verma and Babu (1995). Fluorescent *in situ* hybridization (FISH) was carried out by means of biotinylated 18S rDNA probes (segment with 1700 pb) obtained from the nuclear DNA of the fish *Oreochromis niloticus* (Linnaeus, 1758)labeled with biotin-14-dATP by nick translation (Gibco cat Nº 18247-015), according to the manufacturer’s instructions. The hybridization technique, post-hybridization washes and visualization were carried out as reported by Swarça et al. (2001). The chromosomes were classified according to their arm ratios as metacentrics (m), submetacentrics (sm), subtelocentrics (st), and acrocentrics (a) (Levan et al. 1964, Guerra 1986) with modifications. The m, sm, st were considered as biarmed chromosomes and acrocentrics were considered as uniarmed chromosomes.

## Results

**Figure 1.**
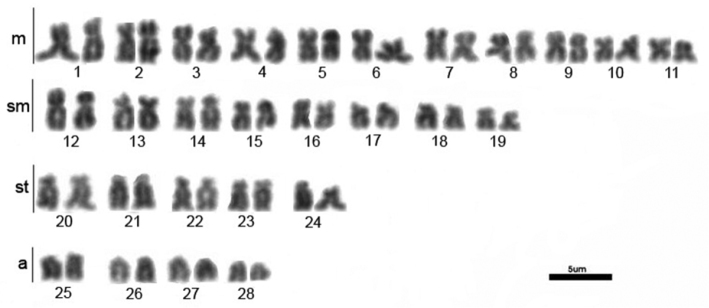
Karyotype of *Hemisorubim platyrhynchos*. Bar = 5mm.

All three populations of *Hemisorubim platyrhynchos* presented the same results. The diploid number obtained was 2n=56 and the karyotype was composed of 22m+16sm+10st+8a (NF=104) without chromosomal differences between sexes (Fig. 1).

**Figure 2.**
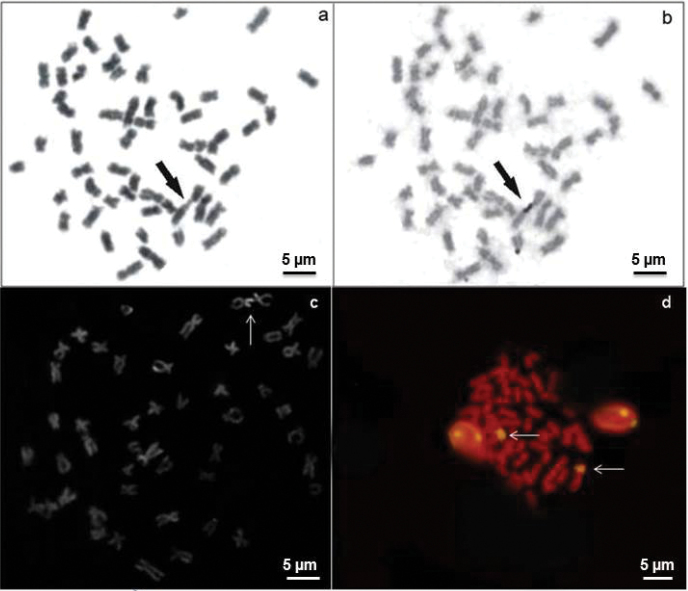
Metaphases of *Hemisorubim platyrhynchos* showing sequential Giemsa-AgNO_3_ staining (**a, b**) CMA_3_ banding (**c**) FISH with 18S rDNA probe (**d**). Arrows indicate the NOR- bearing chromosomes.

**Figure 3.**
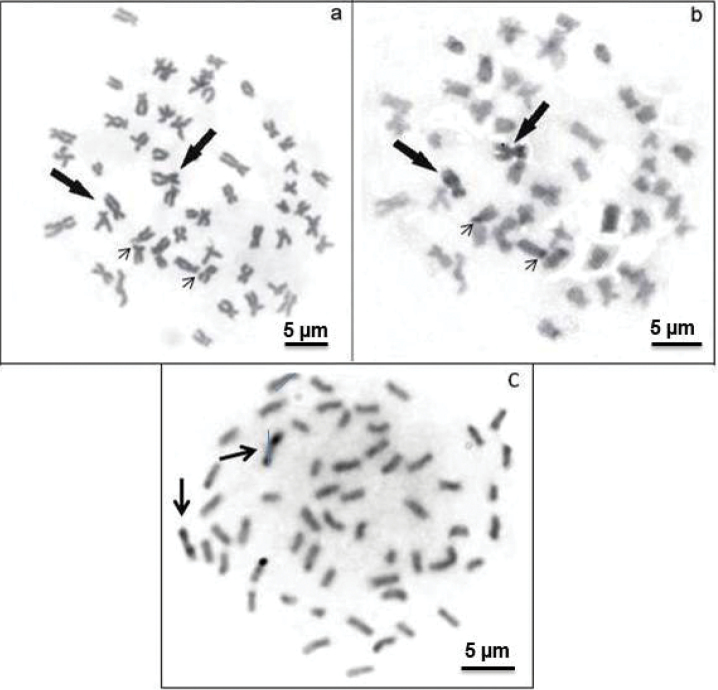
Metaphases of *Hemisorubim platyrhynchos* showing sequential Giemsa-C banding (**a, b**) and after *Alu*I treatment (**c**). The arrows indicate the biarmed chromosome pair (marker) with positive bands on the short and long arms, the thin arrows show the NOR-bearing chromosomes.

The AgNORs were located in the terminal position on the short arm of a subtelocentric (st) pair (Figs 2a, b). The bright signals correspond to the zones evidenced by argentic impregnation after FISH with the 18S rDNA probe and staining with CMA_3_ (Figs 2c, d). This chromosome pair is clearly identified due to its size, shape and exclusive secondary constriction. C-banding revealed positive bands in the pericentromeric regions of some chromosome pairs and on the short arms of a st chromosome pair, coincident with positive C-bands and allowed the identification of a large biarmed marker pair with positive bands on almost the entire short and long arms (Fig. 3b). The *Alu*I restriction enzyme shows bands that resemble C-banding, principally on the biarmed chromosome, producing a reverse pattern (Fig. 3c). The mentionedchromosome could be considered a species-specific cytogenetic marker, since it has not been reported in other species of this group of fish.

## Discussion

The karyotype of *Hemisorubim platyrhynchos* was composed of 22m+16sm+10st+8a (NF=104), however, despite having the same diploid chromosome number 2n=56, *Hemisorubim platyrhynchos* from the Paraná River/Brazil reported by Martins-Santos et al. (1996) presented 22m + 18sm + 6st + 10a (NF=102, recalculated in the present paper). These variations could be ascribed to chromosome rearrangements, although when alternatively using parsimony criteria, differences could be attributed to the condensation of the chromosome arms and/or to technical artifacts. In the family Pimelodidae, 23 of the 27 karyotyped species have a diploid number of 2n= 56 chromosomes, except for *Calophysus macropterus* Lichtenstein, 1819, *Luciopimelodus pati* (Valenciennes, 1840), and *Pinirampus pirinampu* (Spix & Agassiz, 1829) with 2n=50 and *Megalonema platanum* (Günther, 1880)** **with 2n=54, which seem to share other characteristics (Swarça et al. 2007).

One point worth emphasizing is the homogeneity of the karyotypes of species belonging to the “Sorubiminae group” with a clear prevalence of biarmed chromosomes, showing a high fundamental number. A cytogenetic feature shared by all species of this group is the AgNORs localized in the terminal position on the short arm of one pair of st/a chromosomes that also could be evidenced by C-banding, as observed in the present study and in other studied species, such as *Sorubim lima* (Bloch & Schneider, 1801)(Fenocchio and Bertollo 1992, Martins-Santos et al. 1996), *Pinirampus corruscans* (Spix & Agassiz, 1829) and *Pinirampus tigrinus* (Valenciennes, 1840)(Fenocchio and Bertollo 1992, Martins-Santos et al. 1996, Swarça et al. 2005a), *Zungaro zungaro* (Humboldt, 1821) (Martins-Santos et al. 1996, Swarça et al. 2001), *Steindachneridion scriptum* (Miranda-Ribeiro, 1918) (Swarça et al. 2005b)and *Steindachneridion* sp. (=*melanodermatum*) (Garavello, 2005) (Swarça et al. 2006).

CMA_3_ staining and FISH with 18S rDNA exhibited fluorescent signals that correspond to the AgNOR sites (Fig. 2c, d). This correspondence between AgNORs, C-banding, FISH and CMA_3_ staining has already been observed in almost all species of the Pimelodidae family (Swarça et al. 2001, 2008).

The relatively low amount of heterochromatin in chromosomes of *Hemisorubim platyrhynchos* andin other species of the Pimelodidae catfishes suggests that this may be a characteristic of this family. On the other hand, C-banding allowed the identification of a large biarmed pair with positive bands on almost the entire short arm and on the long arm. The *Alu*I restriction enzyme onfish chromosomes produces a C-banding-like pattern (Maistro et al. 2000) and this was also observed in *Hemisorubim platyrhynchos* chromosomes (Fig. 3c). This chromosome pair could be considered a species-specific cytogenetic marker, as it has not been reported in other Pimelodidae (Fig. 3b).

According to cytogenetic traits, this family could be divided into two: the “*Pimelodus* group” and the “Sorubiminae group” (=Sorubinae), and the cytogenetic data confirm that the analyzed species belongs to the second group, because it has 2n=56 chromosomes, a high NF and the NORs localized on one single chromosome pair in the terminal position of the short arms, as it occurs with the other species of this group (Swarça et al. 2007). Thus, despite its wide geographic distribution, it is evident that *Hemisorubim platyrhynchos* shows a marked conservatism in its basic karyotype macrostructure, differing from many species of the Pimelodidae family, i.e., the “*Pimelodus* group”, which presents a wide karyotypic variability, even within the same hydrographic basin and within the same river.
